# Uncovering genetic diversity and admixture of British Africans with HLA alleles inferred from whole genome sequencing

**DOI:** 10.1038/s41431-025-01888-9

**Published:** 2025-07-16

**Authors:** Yunjia Liu, Ze Meng, Indra Adrianto, Albert M. Levin, Qing-Sheng Mi, Qiang Wang, Hongsheng Gui

**Affiliations:** 1 Psychiatry Research and Behavioral Health Services, Henry Ford Health, Detroit, MI USA; 2 Center for Cutaneous Biology and Immunology Research, Department of Dermatology, Henry Ford Heath, Detroit, MI USA; 3 Center for Bioinformatics, Department of Public Health Sciences, Henry Ford Health, Detroit, MI USA; 4 Department of Medicine, College of Human Medicine, Michigan State University, East Lansing, MI USA; 5 Henry Ford Health + Michigan State University Health Sciences, East Lansing, MI 48824 USA; 6 Immunology Research Program, Henry Ford Cancer Institute, Henry Ford Health, Detroit, MI USA; 7 Department of Epidemiology and Biostatistics, College of Human Medicine, Michigan State University, East Lansing, MI USA; 8 Cancer Biology Graduate Program, School of Medicine, Wayne State University, Detroit, MI USA; 9 Department of Biochemistry, Microbiology, and Immunology, School of Medicine, Wayne State University, Detroit, MI USA; 10 Department of Internal Medicine, Henry Ford Health, Detroit, MI USA; 11 Department of Dermatology, Henry Ford Health, Detroit, MI USA; 12 Mental Health Center and Psychiatric Laboratory, West China Hospital of Sichuan University, Chengdu, Sichuan China; 13 Center for Health Policy and Health Services Research, Henry Ford Health, Detroit, MI USA; 14 Department of Psychiatry, Michigan State University, East Lansing, MI USA

**Keywords:** Population genetics, Genome evolution, Haplotypes, Next-generation sequencing

## Abstract

The human leukocyte antigen (HLA) region is highly diverse and plays a crucial role in immune regulation and antigen presentation. Accurate HLA typing is essential for understanding disease susceptibility, transplantation compatibility, and pharmacogenetics. However, its application in African descent populations is challenging due to complex linkage disequilibrium patterns and the lack of ancestry-matched populations in HLA reference panels. Here, we leveraged the latest whole-genome sequencing (WGS) data from UK Biobank African individuals to perform better HLA genotyping, and further utilized allelic and haplotypic data to explore population genetics patterns of this region. With WGS-inferred HLA alleles, we identified specific admixture patterns (predominant West and East African and minor European ancestries) within British African population, revealing their complex evolutionary history. Not only did we reveal the genetic diversity within this population, but also highlighted its differences from African Americans, ancestral Africans, and other global populations. We further identified regional ancestry differences in the HLA genomic region, highlighting discordance between global and local admixture estimates. British Africans also presented unique HLA frequency distributions for both typical and disease-associated alleles or haplotypes. These findings emphasize the need for expanding African-specific HLA reference panel and prove better HLA typing can be achieved by coupling sequencing technologies with computational approaches. The HLA genetic characteristics observed in British Africans provide valuable insights into population-specific immune responses and susceptibility. Overall, this study advances our understanding of HLA diversity and genetic admixture in British African population, with important implications for both disease mechanism and clinical utility.

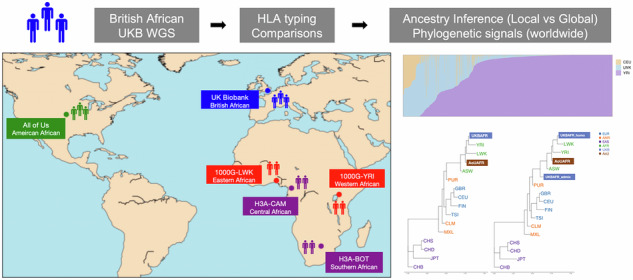

## Introduction

The HLA (commonly referred to as the Human Leukocyte antigen) region, known as the human major histocompatibility complex (MHC), is located on chromosome region 6p21 and contains highly polymorphic genes [1, 2]. The class I (e.g., *HLA-A*, *HLA-B* and *HLA-C*) and class II genes (e.g., *HLA-DPA1*, *HLA-DPB1*, *HLA-DQA1*, *HLA-DQB1*, and several *HLA-DR genes*) encode HLA proteins, which functionally present antigenic peptides to T cells and shape immune responses. The newly reported 42,583 distinct alleles (IPD-IMGT/HLA Release, version 3.60, 2025-04) [3, 4] within this region play a critically important role in the immune response and pathogenesis of auto-immune associated diseases [5, 6]. Significant HLA-disease associations have been identified at various levels, including single nucleotide polymorphisms (SNP), allelic haplotypes, gene expression, and amino acid differences. Moreover, HLA associated SNP genotyping and allele typing have been widely used in immunologic drug reactions [7] and organ transplantation [8]. Revealing deeper insights into this genomic region would facilitate a breakthrough comprehension of the underlying pathological mechanisms in multiple diseases with unclear immunologic etiology. The high polymorphism of the MHC region also makes it useful in the anthropologic tracing of human population migration [9], implying that allelic variations in HLA genes tend to be population-specific.

Due to its structurally complex and highly polymorphic nature [10, 11], and long-term natural selection [12], it is a daunting task to identify the functional allele and fine-mapping causal variations. Historically, HLA typing methods have evolved from serology-based techniques to sequence-specific primer polymerase chain reaction (PCR) and oligonucleotide probes, and subsequently advanced to Sanger sequencing, which remains time-consuming and expensive [13]. Amplicon-based sequencing allows targeted amplification of specific exons or full-length HLA genes and provides high accuracy, but requires dedicated laboratory workflows and is often applied in clinical or small-sample research setting. HLA imputation has become a popular and cost-effective alternative approach to traditional HLA typing methods [14]. Despite the progress in population-scale HLA reference panel for European and Asian populations [15, 16], African populations have still been underrepresented in the literature [17, 18].

Recent biobank-scale short-read whole genome sequencing (WGS) projects provide new solutions, such as direct calling from assembled or re-aligned sequence reads. Notably, both the UK Biobank (UKB) and All of Us (AoU) project encompass over 1000 African samples with WGS data [19, 20], with representation from a broader range of regions and ethnic groups than previously available. Specifially, genetic admixture and HLA diversity in African Americans have been studied in the CAAPA and TOPMed projects [21, 22]; however, similar research on British Africans remains lacking. This lack of representation hinders our understanding of HLA diversity and its role in disease susceptibility in individuals of African descent.

Leveraging the latest African genomic data, this study has two aims. Firstly, it aims to implement and compare three common methods for identifying HLA allele genotypes based on different genomic data from the UKB and 1000 G. This comparative analysis evaluates the incremental values of WGS data. Subsequently, we will detect genetic diversity and genetic admixture patterns among the UKB population and compare it with other African populations in 1000 G and AoU programs. By leveraging new biobank-scale genomics data and novel HLA tools, our study not only addresses significant analytical gaps in African population HLA research but also reinforces the consideration of ancestry in genetic analyses. These findings will contribute to a deeper understanding of the immunogenetic diversity in African populations and enhance HLA research in global health.

## Material and methods

### Study subjects and existing data

The study subjects in the primary cohort comprised 1,199 individuals (50% females) in the UK Biobank. All individuals were subjects with self-reported African ancestry (UK Biobank Data-field 21000). All these UKB participants underwent both genome-wide genotyping and whole-genome sequencing (Data-field 22418 and 23193) [20, 23], which were used to infer HLA genotype in our analysis. It should be noted that no benchmark HLA genotypes were generated by the traditional gold standard.

For the auxiliary dataset, 100 unrelated African individuals (50% females) were randomly chosen from the 1000 Genomes Project (1000 G). High-quality genotyping and benchmark HLA data (via Sanger sequencing) were available for these 1000 G African samples. The assay genotypes from the 1000 G phase three panel [24] and high-coverage whole-genome sequencing (with a depth of 30X) [25] data were used. To enhance the ethnic diversity of the samples, we also collected genotype from the Human Heredity and Health in Africa (H3Africa) Consortium [26] and the All of Us (AoU) Research Program [27]. More details are included in the Supplementary Methods.

To our knowledge, there is no sample overlap among these cohorts. All individual identifiers and personal information were rendered unidentifiable during the analysis. For subsequent population genetics analysis, the UKB African, AoU African, H3Africa, and 1000 G populations were included. The informed consent and ethics details for the UK Biobank, 1000 Genomes Project, H3Africa and All of Us project was described in the previous publications [23, 24, 26, 27]. The detailed ethnic group code was summarized in Table S1. More details about the above data processing were also described in the corresponding publications [19, 20, 23–26]. Meanwhile, the overview of our study design was showed in Figure S1.

### Three HLA allele typing methods for African population

In the comparisons among HLA typing methods, we restricted our analysis to subjects with both genotype and whole genome sequencing data available in the two cohorts (UKB and 1000 G). Overall, we selected three methods for HLA genotyping for African individuals in the UK Biobank: (i) 3-field HLA genotypes directly called from whole genome sequencing using HLA*LA (‘linear alignments’) [28], a novel graph-based method for HLA type inference; (ii) imputation from assay genotypes using the imputation software Minimac4 within the Michigan imputation server (MIS) [29], which is well-recognized and widely used in the HLA imputation; (iii) imputation using HLA*IMP:02 [30] which was provided by the UKB (IMP:02) and is not publicly accessible. More details about the above three methods were included in the Supplementary Methods.

We first examined the number of unique alleles observed at each *HLA* locus. Specifically, we compared the total number of unique alleles with those having frequencies below 0.05 and 0.01, respectively. Then, we compared the genotype results obtained from the three methods in pairs, considering that there was currently no gold standard genotype reference for UKB participants. We restricted our comparisons and further statistics to the HLA typical genes including 3 for class I (*HLA-A*, *B*, *C*) and 5 for class II (*HLA-DQA1*, *DQB1*, *DRB1*, *DPA1*, *DPB1*). The concordance was calculated at both first field and second field resolutions, by dividing the number of matching genotypes (based on truncated HLA nomenclature) identified through the two methods by the total number of individuals. Furthermore, a sensitivity analysis was conducted on another group of African samples from 1000 G with an available gold standard genotype. We utilized the first two methods, HLA*LA and MIS. Due to the limited number of loci in classical Sanger sequencing (Sanger), we then compared its results with the five-locus HLA genotype, including *HLA-A*, *HLA-B*, *HLA-C*, *HLA-DRB1*, and *HLA-DQB1*.

### Ancestry estimation and phylogenetic signals in UK Biobank African populations

To assess global ancestry and admixture patterns, we initially performed an unsupervised ADMIXTURE [31] (version 1.3.0) analysis with the number of subpopulations (K) ranging from 1 to 8. The optimal number of ancestral reference groups was selected by cross-validation (CV) error. In the supervised ADMIXTURE analysis, we incorporated 1000 G populations (Yoruba in Ibadan from Nigeria, YRI; Luhya in Webuye, Kenya, LWK; Utah residents with Northern and Western European ancestry, CEU) and H3Africa populations (Botswana, BOT; Cameroon, CAM) as reference groups. The markers across the whole genome were selected based on the SNP list from HapMap3 and pruned by the PLINK [32] “--indep-pairwise 50 10 0.01” command. Various combinations of genetic ancestry compositions were assessed and detailed in the Supplementary Methods. Then, we dissected the ancestry proportions of UKB Africans using CEU, LWK and YRI groups from 1000 G.

For the local ancestry inference based on the MHC (GRCh37, chromosome 6, BP: 28,477,797–33,448,354) region, 1,198 UKB array genotypes were phased using Beagle (version 5.4) [33] and merged with the above 1000 G African and European genomes. Using random forest discriminative methods and conditional random field model, the RFMix (version 2.0) [34] inferred the local ancestry of multiple segments within the MHC region with default options. Based on the local ancestry inference of these segments, we calculated the ancestry proportions from the MHC region and assessed the correlation with global ancestry proportions. As a sensitivity analysis, we randomly selected five genomic regions of the same length, seen in the Supplementary Method. Moreover, we set a threshold of 0.2 for the European ancestry (CEU) proportion, categorizing UKB African individuals into a homogeneous subgroup (African ancestry proportion >0.8) and an admixed subgroup (European ancestry proportion >0.2). Sensitivity analysis was performed using alternative ancestry thresholds (European ancestry >0.1 or >0.3), detailed in the Supplementary Methods.

To pinpoint the HLA diversity in the UKB African population and its two subgroups, we compared our second field genotype with the five-locus Sanger genotype of fourteen worldwide representative ethnic groups from 1000 G. Additionally, we included a dataset of 983 AoU African samples, where the HLA genotypes were obtained using the Kourami [35] software. The five-locus HLA allele frequencies from these populations were used to estimate genetic distance and construct a phylogenetic tree. The Nei’s standard genetic distance (***D***_***ST***_) and the resulting phylogenetic tree using the Neighbor-Joining (N-J) method [36] were implemented in the POPTREE2 software [37]. The bootstrap test for the N-J tree was conducted with 5000 iterations.

### Population genetics analysis within UKB African populations

High-resolution (second field) HLA genotypes, which were extracted from HLA*LA typing results, were analyzed in the Python for Population Genomics (PyPop, version 1.0.0) software [38]. Allele counts and frequencies, Hardy-Weinberg equilibrium proportions (HWP) test and Ewens-Watterson homozygosity (EWH) test of neutrality [39, 40] were performed in the UKB African population and two subgroups, separately. For each pair of loci, all pairwise linkage disequilibrium (LD) was estimated, including two overall LD measures (Hedrick’s statistic ***D’*** [41] and Cramer’s V statistic ***W***_***n***_ [42]) and conditional asymmetric LD (cALD) measures (***W***_***A/B***_ and ***W***_***B/A***_) [43]. Due to multiallelic loci, cALD would capture the heterogeneity in genetic variation and facilitate a more precise correlation between two HLA loci. Moreover, haplotype frequencies were then estimated using the expectation–maximization (EM) algorithm. We also cross-checked the frequencies of common HLA genotype and haplotypes identified in UKB across African populations from the Allele Frequency Net Database (AFND) [44] and the Anthony Nolan register [45]. Additionally, to illustrate the clinical importance, we also searched the Pharmacogenomics Knowledgebase (PharmGKB) [46] for drug associations with the common HLA genotypes.

## Results

### HLA*LA typing results and comparisons

For the UKB African populations, we inferred HLA genotypes using three different methods: HLA*LA, MIS, and IMP:02. Across most HLA loci, HLA*LA consistently identified the highest number of unique genotypes, both in total (*N* = 292) and rare alleles (<0.05, *N* = 242; <0.01, *N* = 182). In contrast, IMP:02 reported the fewest genotypes (total, *N* = 195; <0.05, *N* = 146; <0.01, *N* = 90). Full counts for each locus and frequency threshold are provided in the Table S2. We calculated the concordance and cautiously compared the genotypes from these three methods in pairs among UKB participants (*N* = 1195, 4 were excluded because the genotype was not provided by HLA*IMP:02), as shown in Table 1 and Table S3. Notably, overall, the HLA*LA genotypes were comparable to the MIS genotypes, while the IMP:02 genotype showed a distinct difference from the other two. For each gene, the biallelic first field and second field concordance rates were highest for *HLA-A* (first field: 91.05–97.07%; second field: 87.78–94.14%), but there was a decline in these rates observed for *HLA-B* (first field: 62.26–92.55%; second field: 58.16–89.21%) and *HLA-DPB1* (first field: 68.45–92.72%; second field: 67.28–92.47%). This level of consistency was notably lower than that for the Europeans and should be approached with caution when using imputed HLA genotypes directly for African samples in the UKB. For the sensitivity analysis in the 1000 G samples (Tables S4-5), the biallelic first field concordance was 98–100% for all genes in a limited sample size. However, for the second field concordance, it was much lower for *HLA-DQA1 (71*–*100%)* but moderate for the other genes (88–100%).

**Table 1:** Comparisons of four-digit HLA typing genotypes in the UK Biobank African populations.

HLA locus	IMP:02 vs MIS			IMP:02 vs HLA*LA			MIS vs HLA*LA		
	Proportion of two match	Proportion of one match	Proportion of no match	Proportion of two match	Proportion of one match	Proportion of no match	Proportion of two match	Proportion of one match	Proportion of no match
HLA-A	88.87%	10.46%	0.67%	87.78%	11.88%	0.33%	94.14%	5.52%	0.33%
HLA-B	58.16%	36.32%	5.52%	58.83%	36.15%	5.02%	89.21%	10.54%	0.25%
HLA-C	77.74%	20.17%	2.09%	76.74%	20.84%	2.43%	91.38%	8.12%	0.50%
HLA-DPA1	63.18%	31.63%	5.19%	62.93%	28.03%	9.04%	83.68%	10.38%	5.94%
HLA-DPB1	67.28%	27.78%	4.94%	67.62%	27.87%	4.52%	92.47%	7.36%	0.17%
HLA-DQA1	81.26%	17.49%	1.26%	82.01%	16.74%	1.26%	98.83%	1.17%	0.00%
HLA-DQB1	67.70%	28.95%	3.35%	67.87%	28.87%	3.26%	96.74%	3.18%	0.08%
HLA-DRB1	70.96%	26.03%	3.01%	72.38%	24.52%	3.10%	91.21%	8.45%	0.33%
Overall	71.89%	24.85%	3.25%	72.02%	24.36%	3.62%	92.21%	6.84%	0.95%

*IMP:02* HLA alleles imputed by HLA*IMP:02 software, data as provided by UK Biobank itself, *MIS* Michigan Imputation Server, HLA*LA HLA*LA (linear alignment).

### Global and local patterns of genetic ancestry substructure

**Fig. 1:**
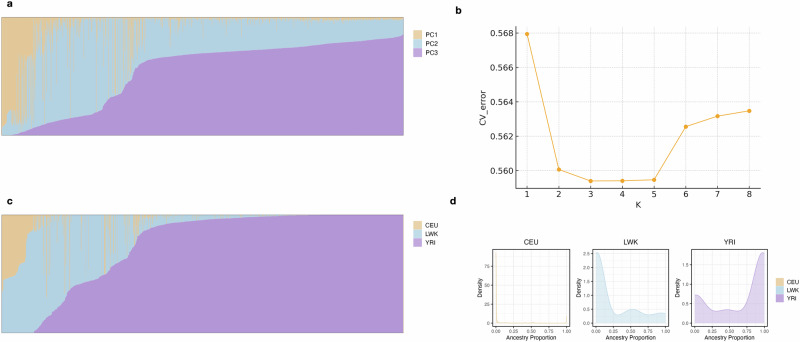
The global admixture pattern of ancestry proportions using ADMIXTURE analysis in the UK Biobank African populations. **a** Estimates of global ancestry of the UK Biobank African populations using unsupervised ADMIXTURE analysis (K = 3), PC 1-3, principal component 1-3; **b** The plot of Cross-validation (CV) error in the unsupervised ADMIXTURE analysis from K = 1 to 8; **c** Estimates of global ancestry of the UK Biobank African populations using supervised ADMIXTURE analysis on 3 ancestral reference populations (YRI, Yoruba in Ibadan from Nigeria; LWK, Luhya in Webuye, Kenya; CEU, Utah residents with Northern and Western European ancestry); **d** the density distribution plot of three ancestry proportions in the supervised ADMIXTURE analysis on 3 ancestral reference populations (YRI, LWK and CEU).

For global ancestry, the results of both unsupervised and supervised ADMIXTURE analysis were displayed in Fig. 1. According to the lowest CV error, the optimal number of unsupervised ADMIXTURE analysis was 3 in the UKB African populations. To provide a broader view of the clustering, we presented the results for K = 2 through K = 8 in Fig. S2. In the supervised analysis, we selected combinations of diverse genetic ancestry components as the reference group, as seen in Figs. S3-6. We found that the African ancestry primarily originated from East Africa and West Africa, represented by the LWK and YRI groups, respectively. Including the CEU group representing Europe, we used these three populations as references for the supervised ADMIXTURE analysis. On average, the UKB African individuals were 94.7% African (with 74.6% inferred from the YRI group and 20.1% inferred from the LWK group) and 5.3% European inferred from the CEU group.

**Fig. 2:**
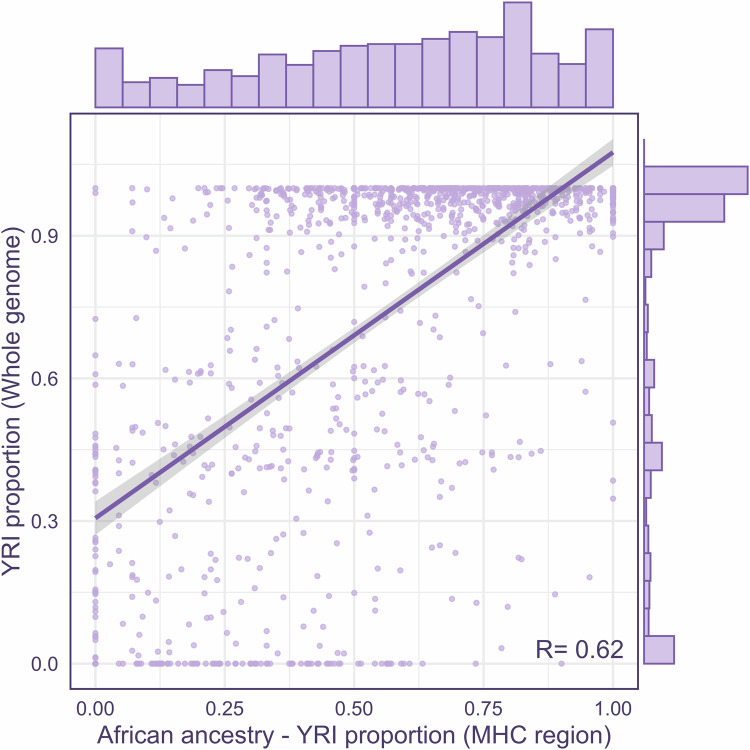
Proportional association of African ancestry between whole genome and MHC region. African ancestry—YRI proportion (MHC region) estimated by RFmix2 software based on the MHC region; YRI proportion (Whole genome) estimated by ADMIXTURE analysis across whole genome; the histogram on the x-axis and y-axis shows the distribution of YRI proportions in the MHC region and whole genome, respectively. The correlation coefficient (R) between the two proportions is 0.62; YRI, Yoruba in Ibadan from Nigeria; MHC, Major Histocompatibility Complex.

The local ancestry inference on the MHC region offered a granular perspective and revealed a region-specific pattern. A total of 226 genetic segments were used for local ancestry inference. The local ancestry proportion based on the MHC region was calculated and then utilized to categorize UKB African populations into a homogeneous subgroup (UKBAFR_homo, *N* = 1006) and admixed subgroup (UKBAFR_admix, *N* = 192). Meanwhile, the main African ancestry proportion, inferred by the YRI group, was highly correlated between estimates at the global and local levels (r^2^ = 0.62, *P* = 2.2 × 10^−16^), as shown in Fig. 2. And correlation coefficients for all five randomly selected regions were higher than that of the MHC region in the sensitivity analysis, as shown in Fig. S7.

### Natural selection, and phylogenetic signals in the MHC region

To investigate HLA evolution, Slatkin’s EWH test was implemented for the HLA loci in the UKB African population and the homogeneous group (Tables S9 and S13). The homozygosity statistic (*F*) was calculated as the sum of the squared allele frequencies, which denotes the observed homozygosity. All the normalized deviates of *F* (*F*_*nd*_) were negative for the above HLA loci, except for *HLA-DPB1*. Negative *F*_*nd*_ values indicate balancing selection, which is expected to increase the number of intermediate frequency variants [12]. For the homogeneous group, a significant negative *F*_*nd*_ value was observed only for the *HLA-DQA1* locus(*F*_*nd*_ = −1.726, *P* = 0.0028) indicating balancing selection.

**Fig. 3:**
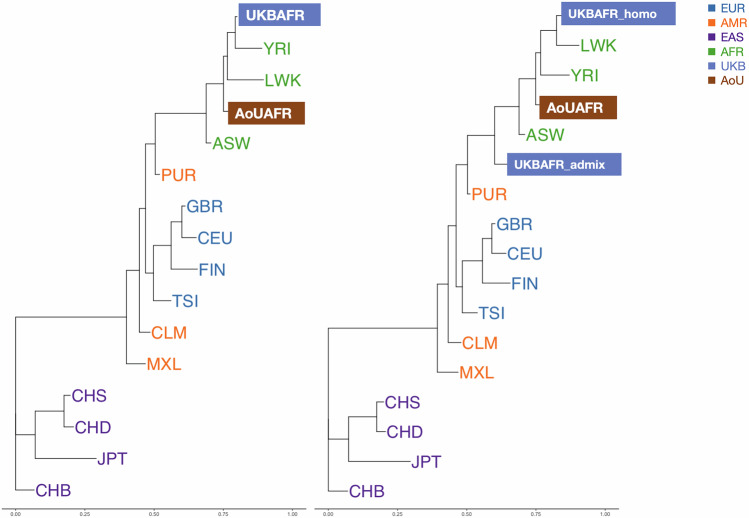
Phylogenetic tree for UK Biobank African population and other worldwide populations. Left: N-J phylogenetic tree among 1000 G worldwide populations, UK Biobank (UKB) and All of Us (AoU) African populations (UKBAFR and AoUAFR). Right: N-J phylogenetic tree among 1000 G worldwide populations, UKB African homogenous subgroup (UKBAFR_homo), UKB African admixed subgroup (UKBAFR_admix), and AoU African populations (AoUAFR). 1000G populations: LWK(Luhya from Webuye,Kenya), YRI(Yoruba from Ibadan, Nigeria), ASW (African Ancestry from Southwest, USA), CLM (Colombian from Medellin, Colombia), MXL (Mexican Ancestry from Los Angeles-California, USA), PUR (Puerto Rican, Puerto Rico), CHB (Han Chinese from Beijing, China), CHD (Chinese from Denver-Colorado, USA), CHS (Han from south, China), JPT (Japanese from Tokyo, Japan), CEU (Northern and Western European from Utah, USA), FIN (Finnish, Finland), GBR (British from England and Scotland, UK), TSI(Italian from Tuscany, Italy).

The pairwise *D*_*st*_ matrix and the N-J phylogenetic tree were estimated based on the five-locus HLA genes from the above UKB, AoU and 1000 G datasets (Fig. 3; Table S6). The phylogenetic tree showed the genetic diversity and evolutionary relationships based on the MHC region. The UKB and AoU African population both shared a common ancestry with the three representative African groups of 1000 G, yet distinct genetic affinities were evident among them. The AoU African population was closer to the ASW (African Ancestry in Southwest US) group, while the UKB African population was closer to YRI (Yoruba in Ibadan, Nigeria). After dividing into two subgroups, the UKB African homogeneous subgroup exhibited a greater genetic similarity to the LWK (Luhya in Webuye, Kenya) group. Meanwhile, the UKB African admix subgroup was located closer to European and American ancestry. The results remained consistent across alternative ancestry thresholds (Figs. S8-9).

### HLA allele frequencies and linkage disequilibrium

We presented the population genetics characteristics of both UKB African populations and the homogeneous group in Tables 2 and S7-14. Based on the HLA*LA inferred genotypes, a total of 292 distinct alleles across eight HLA loci were identified in the UKB African population, while 257 alleles were identified in the homogenous group. The distributions of the HLA class I (*HLA-A*, *HLA-B*, and *HLA-C*) and class II (*HLA-DQA1*, *HLA-DQB1*, *HLA-DRB1*, *HLA-DPA1* and *HLA-DPB1*) genotypes were summarized in Table 2. Those common frequencies observed in the UKB African population were compared with the larger sample size of African populations from the AFND and the Anthony Nolan register [45]. Additionally, we included previously reported drug associations from the PharmGKB, all of which are presented in Table S17.

**Table 2:** Comparisons of HLA allele or haplotype frequencies between UKB overall African population (All) and its homogeneous subgroup (Homo).

Locus/Haplotype	Group	>0.1	>0.05	>0.01	>0.005	>0.001	Allele Counts
HLA-A	All	1	9	18	24	37	53
	Homo	1	9	17	21	34	49
HLA-B	All	1	5	21	30	44	76
	Homo	1	8	21	29	36	65
HLA-C	All	2	7	17	18	28	37
	Homo	3	6	16	18	21	35
HLA-DQA1	All	5	6	7	7	7	8
	Homo	4	6	7	7	7	7
HLA-DQB1	All	4	5	11	12	15	21
	Homo	4	5	11	12	15	19
HLA-DRB1	All	1	9	19	22	26	42
	Homo	1	9	18	21	23	34
HLA-DPA1	All	3	4	6	7	11	13
	Homo	4	4	6	7	10	13
HLA-DPB1	All	3	5	11	15	22	42
	Homo	3	5	11	13	22	35
Locus Overall	All	20	50	110	135	190	292
	Homo	21	52	107	128	168	257
HLA-B ~ HLA-C	All	1	4	23	40	96	217
	Homo	1	3	24	37	81	183
HLA-DPA1 ~ HLA-DPB1	All	3	5	12	19	44	87
	Homo	3	5	13	19	40	75
HLA-DRB1 ~ HLA-DQA1 ~ HLA-DQB1	All	1	5	27	33	59	111
	Homo	1	5	23	31	56	92

All, all African populations in UK Biobank. Homo, the homogenous subgroup from all African populations in UKB. The subgroup was determined by local ancestry proportions estimated from RFmix2.

For each locus, the observed genotype counts were compared to those expected under Hardy Weinberg proportions (HWP), using Guo and Thompson’s exact method. The Hardy-Weinberg equilibrium (HWE) deviations and heterozygosity index were shown in Tables S9 and S13. For the homogenous subgroup, only two genes deviated from HWE expectations, including *HLA-DPA1* (*P* < 0.0001) and *HLA-DPB1* (*P* = 0.0016). However, in the whole population, there were two additional genes with deviation from HWE, specifically *HLA-B* (*P* = 0.0003) and *HLA-DRB1* (*P* = 0.0237).

To display the co-inheritance pattern between HLA loci, the global picture of pairwise linkage disequilibrium (LD) is shown in Fig. S10. All the HLA loci pairs showed significant LD in the UKB African population and the homogeneous subgroup. The pairwise LD was estimated by two overall LD measures (***D’*** and ***W***_***n***_) and cALD measures (***W***_***A/B***_ and ***W***_***B/A***_), which was summarized in Table S10. In the homogeneous group, the strongest biallelic LD was *HLA-DQA1: HLA-DRB1* (*D’* = 0.89), *HLA-DQB1: HLA-DRB1* (*D’* = 0.87), *HLA-DQA1: HLA-DQB1* (*D’* = 0.87), *HLA-B: HLA-C* (*D’* = 0.86) and *HLA-DPA1: HLA-DPB1* (*D’* = 0.83). The lowest value was seen in *HLA-C: HLA-DPA1* (*D’* = 0.186). For the conditional asymmetric LD, the cALD_HLA-B/HLA-C_ and cALD_HLA-C/HLA-B_ was 0.61 and 0.79 respectively, which indicates that there are more variations of *HLA-B* compared to those of *HLA-C*.

Based on the highest pairwise LD, the three haplotype frequencies were estimated (Tables S15-16). The most frequent haplotype in the homogeneous group was HLA-DRB1*15:03 ~ HLA-DQA1*01:02 ~ HLA-DQB1*06:02(15.2%), *HLA-B* *53:01 ~ *HLA-C* *04:01(14.6%), HLA-DPA1*02:02 ~ HLA-DPB1*01:01 (20.4%) and HLA-DPA1*02:01 ~ HLA-DPB1*01:01(19.9%), respectively. We also displayed the common haplotypes frequencies across populations from the AFND [44] in Table S18.

## Discussion

Leveraging the most recent WGS data from the UKB, this study provides a comprehensive genetic analysis to explore the uncovered HLA characteristics of African samples from the UK Biobank. In this work, we firstly evaluated the HLA typing using the latest WGS data and compared it with classic typing methods across different genetic datasets. More notably, we further examined the genetic diversity and admixture patterns within the British African population and compared them with other African populations and worldwide populations. Our findings emphasize the importance of accurate HLA typing in underrepresented populations with complex genetic backgrounds, such as those in the biobank. To further illustrate these patterns, we also provided detailed population genetics metrics among the British African population in the UK Biobank.

We conducted a comparison among the results from three unique methods of HLA typing, including the MIS imputed genotype, the officially provided genotype from the UKB and the HLA*LA genotype. Additionally, we implemented the HLA*LA graph-based alignment on the UKB DNAnexus platform, showing the benefit of cloud-based parallel computation and large-scale storage. Overall, HLA*LA identified more unique genotypes, suggesting this method may be more sensitive in detecting allelic diversity compared to MIS and HLA*IMP:02. One possible explanation is that HLA*LA directly uses sequencing reads, which may allow it to detect rare alleles that are missed by imputation-based methods. These differences in allele detection capacity may affect downstream analyses and show the necessity and advantage of using WGS-called HLA genotypes.

Moreover, the concordance between the HLA*LA and MIS genotypes was comparable at both the first field and second field levels. The concordance of HLA*LA was slightly higher than that of MIS when compared to the IMP:02 genotype. The IMP:02 genotype had notably the lowest concordance, possibly due to the use of old imputation methods. Our result also suggested additional caution is needed when using HLA*IMP:02 provided HLA data for disease association studies involving African populations. The replication analysis from 1000 G also yielded similar trends, although the sample size was limited, and they were part of the widely used reference panels for method development. As for specific loci, *HLA-B* showed notably lower concordance, possibly due to its high polymorphism.

To achieve more accurate HLA typing, it is necessary not only to explore different types of genetic data but also to improve relevant statistical methods [14]. For example, large-scale Whole Exome sequencing (WES) data is accessible and used to call the HLA alleles using HLA-HD algorithm in the UKB [47]. Moreover, amplicon sequencing and long-read WGS data allows for the detection of novel HLA alleles and haplotypes, based on high-resolution assembly [48]. In a recent study, HLA*LA was able to take advantage of long-read data to achieve an average accuracy of 98%, even in highly diverse South African samples [28]. With the enlargement of sample size and qualified high-depth sequencing data, the HLA*LA may have a better performance.

Due to advancements in HLA typing methods, we had the opportunity to explore the comprehensive genetic architecture of these underrepresented African populations, particularly based on the MHC region. First, we dissected the genetic ancestry components at both the whole-genome and MHC region specified solutions. To achieve this, we utilized high-quality African genetic reference datasets, including H3Africa and 1000 G. After evaluating various reference panels, we selected two African populations, Yoruba (YRI) and Luhya (LWK), as well as a European population as the reference groups. Tracing the migration history, the African populations in the UK predominantly originate from countries such as Nigeria, Kenya, and Ghana [49], which aligns closely with the results of our global ADMIXTURE analysis. Moreover, we observed a subtle discrepancy between globally and locally inferred genetic admixture. The high polymorphism of HLA and its critical role in the immune system could contribute to the divergence in genetic structure, which may reflect immune-related selection [5, 6]. These distinct patterns could provide valuable insights into the etiology of complex diseases and transplantation medicine, as well as selection.

Then, to further explore the evolutionary forces at play, we conducted both balancing selection and phylogenetic analyses specifically within the MHC region. Balancing selection was shown for all HLA loci with negative *F*_*nd*_ values, which suggests the effect of human migration, long-term pathogen-driven selection, and diverse population interactions [12, 50]. Moreover, we identified the phylogenetic signals, using two representative biobank-scale genomic datasets from African populations in the UK Biobank and All of Us. These two groups exhibited distinct admixture patterns and varying degrees of relatedness to various classical African and other worldwide populations of 1000 G. The phylogenetic tree may indicate that these biobank-scale African populations provide a reasonable representation of local populations, to a certain extent. However, despite the availability of biobank-scale data, the sample size of African populations remains relatively small, and notable heterogeneity persists. And the evolutionary pattern across the UKB and other populations needs to be studied in the future with more comprehensive approaches (autosome, Y chromosome, and mitochondria) [51]. Additionally, by subdividing the UKB African populations based on ancestry proportions into two distinct groups, we observed unique genetic distances. This highlights the importance of accounting for the admixture structure and underlying heterogeneity in genetic analyses of African population within biobanks, particularly when interpreting disease associations.

Additionally, based on the second field classical HLA genotypes from HLA*LA, we captured the genetic diversity in UKB African samples, particularly the high polymorphism observed in *HLA-B*. The distribution of HLA allele frequencies also highlights the need for deeper exploration of HLA diversity in African populations. These findings are mainly consistent with prior reports on genetic diversity in African populations and emphasize the importance of including diverse ancestries in HLA research [44, 52]. Most of the identified common HLA genotypes are closely associated with drug efficacy or adverse reactions reported in PharmGKB, but further validation is still needed in these underrepresented populations. Such associations are particularly important for precision medicine and healthcare equity, as pharmacogenomics testing is becoming increasingly common in clinical practice.

According to the observed high LD between HLA loci, the common haplotype frequencies were also estimated, which was reported in other studies of USA American African and Brazil Caucasian populations [53, 54]. Some HLA genes did not conform to Hardy-Weinberg equilibrium, maybe due to the genetic diversity and our limited sample size. With large-scale sequencing datasets, we will have greater opportunities to identify a broader range of HLA haplotypes across diverse populations [45]. It also emphasizes the importance of enhancing the HLA genotype references in African populations.

However, there are still some limitations in this study. First, the HLA typing comparisons in the UKB lack a gold standard genotype, although imputation methods have been previously benchmarked and shown to achieve high accuracy [28]. Second, the UK Biobank was a volunteer-based study, which may have enrolled non-representative individuals, particularly in the British African population [23]. This also reminds us to consider the genetic admixture and interpret the results with caution in biobank-scale data, particularly in underrepresented populations. Lastly, the genotypes provided by the UKB have been widely used but are short of older methods and assay-based genotype. Future studies should aim to include more representative samples from diverse African populations, apply advanced statistical techniques to further refine HLA typing, and integrate targeted amplicon typing and long-read sequencing technologies for novel alleles. As 500k whole-genome sequencing data from UK Biobank and both short-read and long-read WGS data from All of Us becomes available, future studies will be able to revisit and refine HLA association analyses with greater resolution and population diversity.

In conclusion, we utilized novel methods and a genetic data source to explore HLA typing and reveal HLA diversity within African populations. The advantage of WGS data enables more comprehensive detection of HLA genotypes. We then characterized the genetic admixture patterns of British African populations, highlighting both the internal heterogeneity and ancestral diversity, as well as their genetic distances from various global populations. These findings provide new insights into the genetic landscape of British African populations, reinforcing the necessity of incorporating HLA diversity and admixture patterns into future pharmacogenomic research and disease association studies.

## Supplementary information

Supplemental_revised_clean

Supplemental Tables

## Data Availability

High-coverage WGS data from the 1000 Genomes Project can be publicly accessed via their designated ftp (http://ftp.1000genomes.ebi.ac.uk/vol1/ftp/). Data access to UK Biobank, H3Africa, and All of Us can be applied through data portal of each provider, respectively. The published article includes all secondary datasets generated or analyzed during this study. All the datasets and software used are summarized in the Table S0.

## Code availability

The code generated from this study is provided through (https://github.com/Yunjia-LIU/HLA_WGS_UKB). Other software used in the analysis: HLA*LA (https://github.com/DiltheyLab/HLA-LA); Kourami (https://github.com/Kingsford-Group/kourami); Michigan Imputation Server (https://imputationserver.sph.umich.edu/index.html); PyPop, (Python for Population Genomics, http://pypop.org); POPTREE2 (https://www.med.kagawau.ac.jp/~genomelb/takezaki/poptree2/index.html); ADMIXTURE(https://dalexander.github.io/admixture/); RFMix2 (https://github.com/slowkoni/rfmix); BEAGLE (http://faculty.washington.edu/browning/beagle/beagle.html).
