# Supplementary material for: Uncovering genetic diversity and admixture of British Africans with HLA alleles inferred from whole genome sequencing
Source: Eur J Hum Genet. 2025 Jul 16;33(8):1057–65. doi: 10.1038/s41431-025-01888-9 (PMC12322232; doi:10.1038/s41431-025-01888-9)
Supplement: Supplementary file 1 — Supplemental_revised_clean [file 41431_2025_1888_MOESM1_ESM.pdf]

Supplemental contents

Index

Figure S1 2
Figure S2 3
Figure S3 4
Figure S4 5
Figure S5 6
Figure S6 7
Figure S7 8
Figure S8 9
Figure S9 10
Figure S10 11
List of Supplemental Tables 12
Supplemental Material and Methods 13
Auxiliary Dataset 1: H3Africa dataset 13
Auxiliary Dataset 2: All of US dataset 13
HLA*LA: direct calling from whole genome sequencing in UKB and 10000G 14
Michigan Imputation Server: imputation from assay genotypes in UKB and 10000G 15
UKB-HLA: imputation using HLA*IMP:02 provided by UKB 15
Kourami: direct calling from whole genome sequencing in All of Us 16
The ADMIXTURE analysis in UKB African population 17
Sensitivity analysis: global and local ancestry proportion correlation in UKB African population 18
Sensitivity analysis: the ancestry proportion threshold in UKB African population 18
Reference 20

Figure S1

Figure S1. The overview of study design.

Category	Sample & Data	Tools & Methods	Evaluation metrics
**HLA typing and comparison**	UKB African (WGS & Array, N=1195)	HLA*IMP:02, HLA*LA, MIS	Concordance (First field & Second Field) Unique Allele counts
	1000G African (WGS & Array, N=100)	HLA*LA, MIS, Sanger (gold standard)	Concordance (First field & Second Field)
**Ancestry, admixture, and phylogeny**	UKB African (Array, N=1998), 1000G (Array, N=239), and H3A (WGS, N=96)	ADMIXTURE (Supervised and Unsupervised)	Global ancestry for whole genome
	UKB African (Array, N=1198), 1000G (Array, N=239), and H3A (WGS, N=96)	RFMix2	Local ancestry for HLA and random regions
	UKB African (AF, N=1198), 1000G (AF, N=1114), and AoU (AF, N=983)	POPTREE2	Genetic distance and phylogenetic tree using HLA alleles
**Allele frequency and linkage disequilibrium**	UKB African (AF, N=1198)	PyPop	Allele and haplotype frequencies, LD, HWE, etc.
	AFND (AF, N=418,581), AN (AF, N=5761), PharmGKB (reported drug associations)	Database search and curation	Frequency comparison for common and risk HLA alleles and haplotypes

UKB, UK Biobank; 1000G, 1000 Genome Project; AoU, All of Us;
WGS, whole genome sequencing; Array, array genotype data; MIS for Michigan imputation server
AF, allele frequency of HLA; AFND, frequency in USA NMDP African American pop 2 (N=416,581) from ANFD database; AN, frequency in African population (n=5761) from Anthony Nolan register

Figure S2

Figure S2. The admixture pattern of ancestry proportions using unsupervised ADMIXTURE analysis in the UK Biobank African populations (from k=2 to k=8).

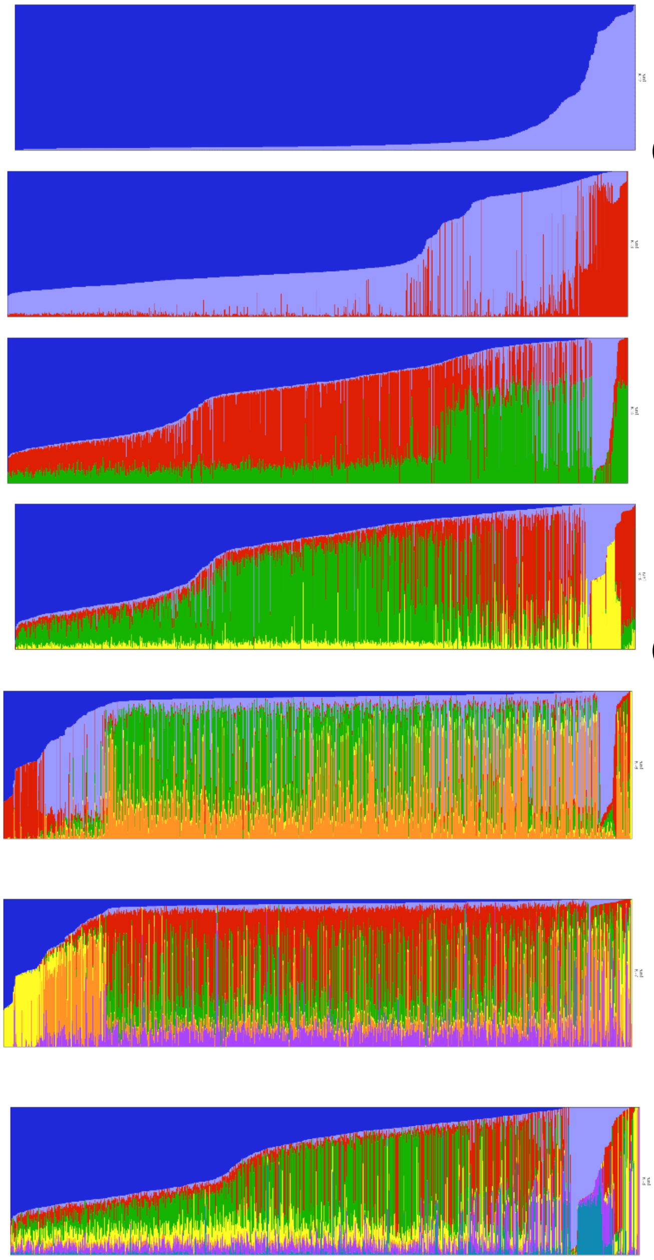

(k=2)

(k=3)

(k=4)

(k=5)

(k=6)

(k=7)

(k=8)

Each plot is the admixture pattern of ancestry proportions (number is the k value) using unsupervised ADMIXTURE analysis in the UK Biobank African populations (from k=2 to k=8)

Figure S3

Figure S3. The global admixture pattern of ancestry proportions using ADMIXTURE analysis in the UK Biobank African populations using four African reference populations.

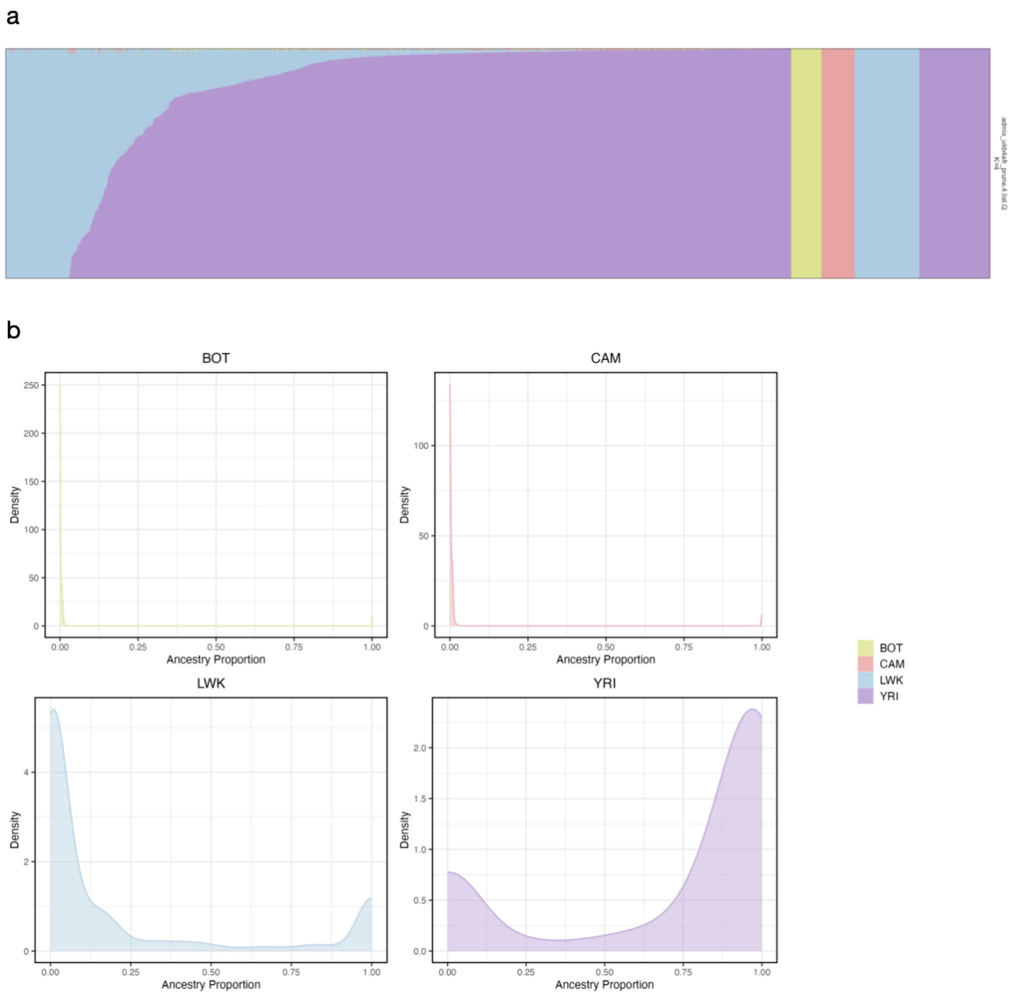

The admixture plot and density distribution of ADMIXTURE analysis using four reference populations: LWK (Luhya in Webuye, Kenya) population representing Eastern Africa and the YRI (Yoruba in Ibadan, Nigeria) population representing Western Africa from the 1000 Genomes Project. Additionally, two populations were selected from the H3Africa dataset: CAM (Bantu and Bantoid Speakers from Cameroon) representing Central Africa and BOT (Multiethnic from Botswana) representing Southern Africa

Figure S4

Figure S4. The global admixture pattern of ancestry proportions using ADMIXTURE analysis in the UK Biobank African populations using two reference populations (LWK, YRI).

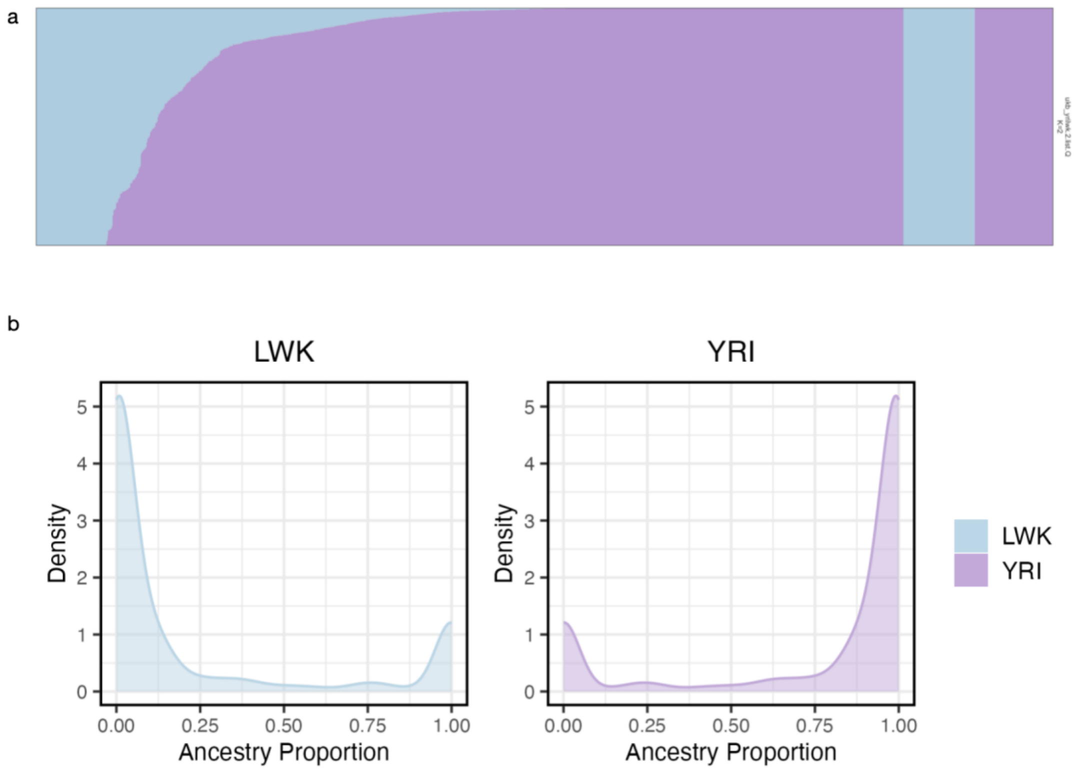

The admixture plot and density distribution of ADMIXTURE analysis using two reference populations: LWK (Luhya in Webuye, Kenya) population representing Eastern Africa and the YRI (Yoruba in Ibadan, Nigeria) population representing Western Africa from the 1000 Genomes Project.

Figure S5

Figure S5. The global admixture pattern of ancestry proportions using ADMIXTURE analysis in the UK Biobank African populations using two reference populations (LWK, CEU).

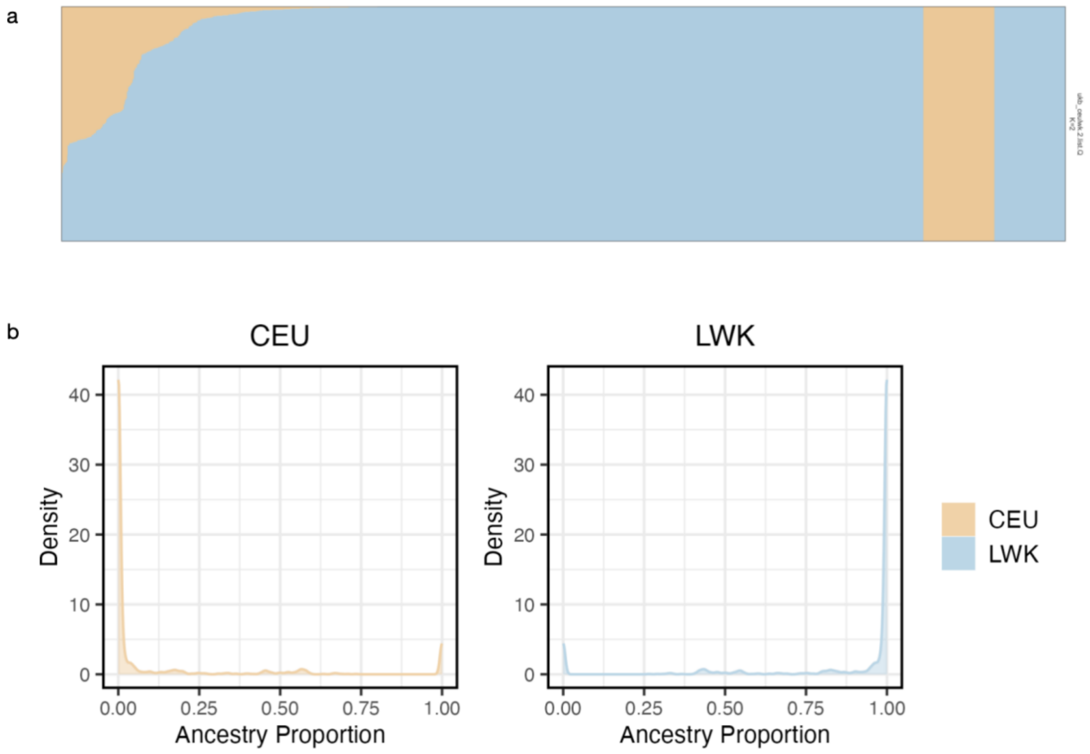

The admixture plot and density distribution of ADMIXTURE analysis using two reference populations: LWK (Luhya in Webuye, Kenya) population representing Eastern Africa and CEU (Utah residents (CEPH) with Northern and Western European ancestry) representing Europe from the 1000 Genomes Project.

Figure S6

Figure S6. The global admixture pattern of ancestry proportions using ADMIXTURE analysis in the UK Biobank African populations using two reference populations (CEU, YRI).

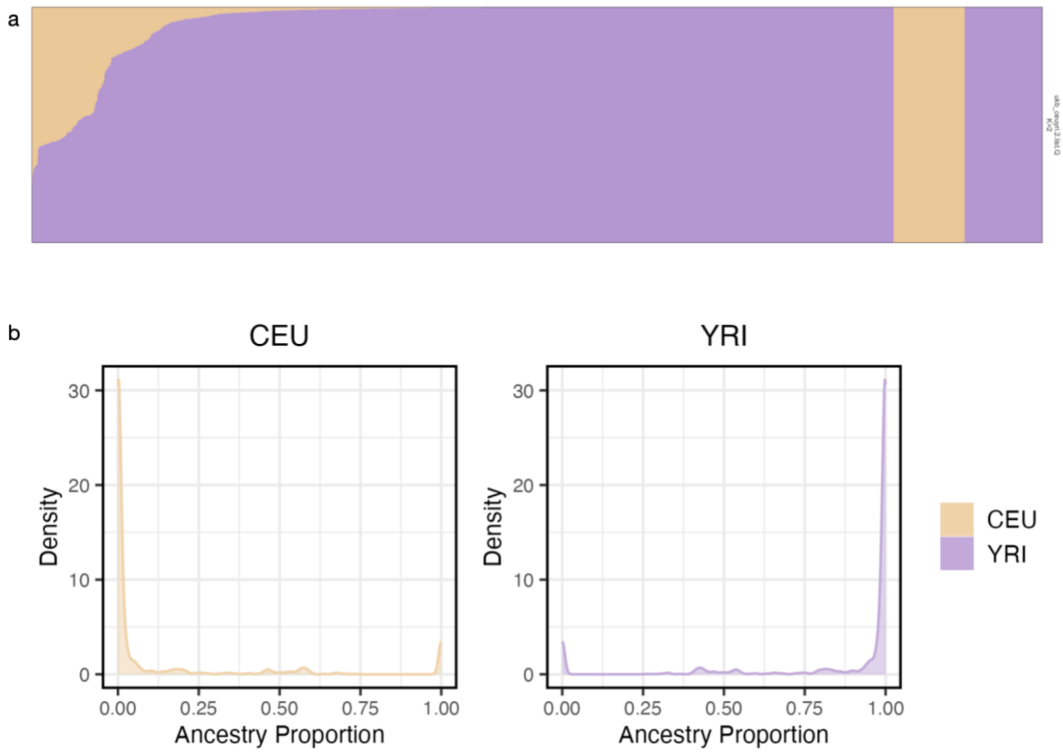

The admixture plot and density distribution of ADMIXTURE analysis using two reference populations: YRI (Yoruba in Ibadan, Nigeria) population representing Western Africa and CEU (Utah residents (CEPH) with Northern and Western European ancestry) representing Europe from the 1000 Genomes Project.

Figure S7

Figure S7. Proportional Association of African Ancestry between Whole Genome and five randomly chosen genomic regions.

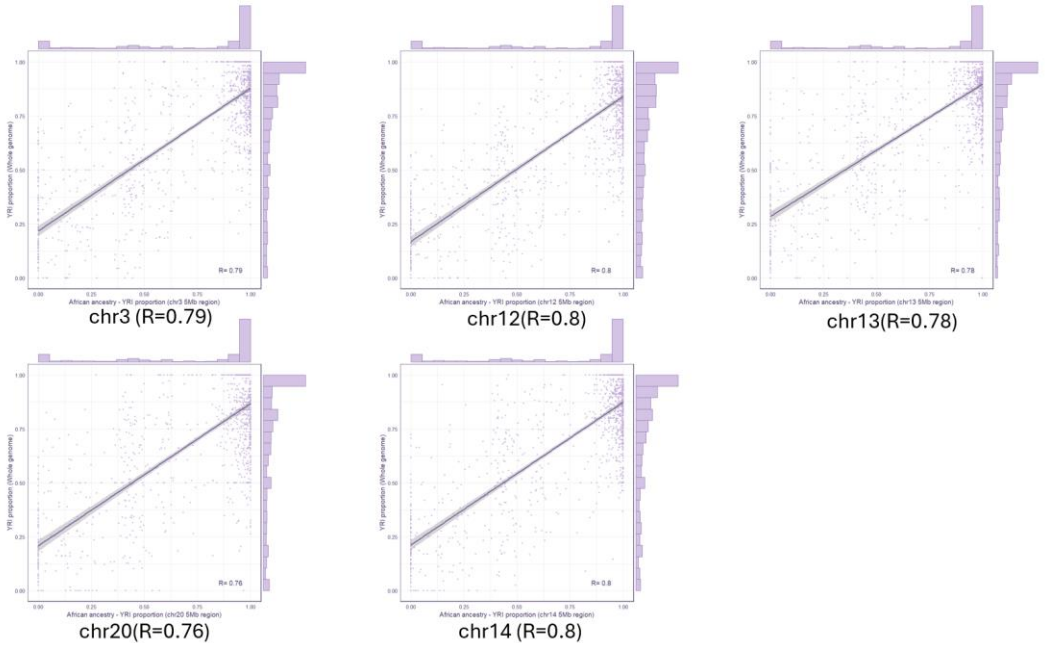

African ancestry – YRI proportion (MHC region) estimated by RFmix2 software based on the MHC region; YRI proportion (Whole genome) estimated by ADMIXTURE analysis across whole genome; the histogram on the x-axis and y-axis shows the distribution of YRI proportions in the randomly chosen genomic region and whole genome, respectively; YRI, Yoruba in Ibadan from Nigeria.

Figure S8

Figure S8. Phylogenetic tree for UK Biobank African population and other worldwide populations (for ancestry proportion threshold 0.3).

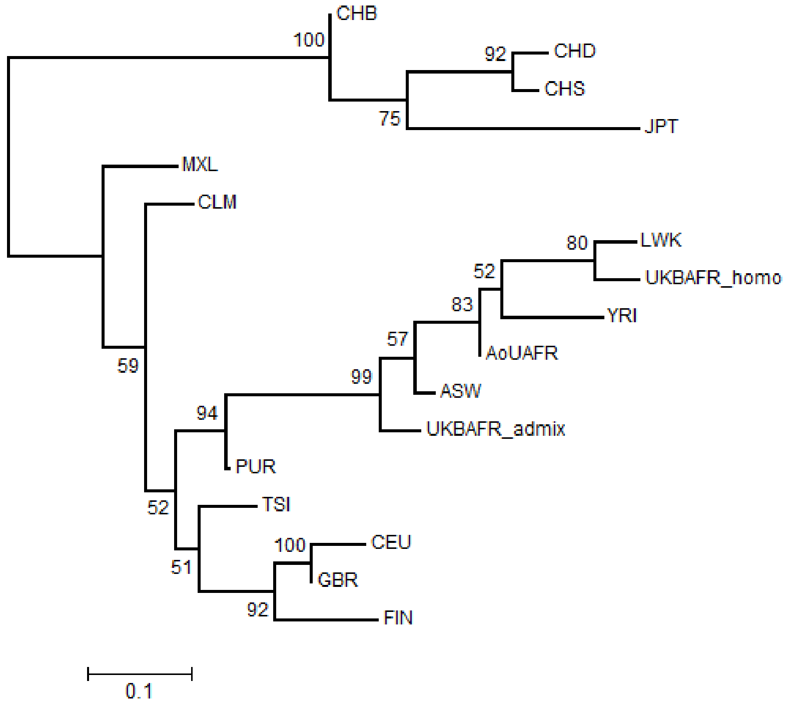

N-J phylogenetic tree among 1000G worldwide populations, UKB African homogenous subgroup (UKBAFR_homo, EUR proportion < 0.3, N=1103), UKB African admixed subgroup (UKBAFR_admix, N=95), and AoU African populations (AoUAFR).

Figure S9

Figure S9. Phylogenetic tree for UK Biobank African population and other worldwide populations (for ancestry proportion threshold 0.1).

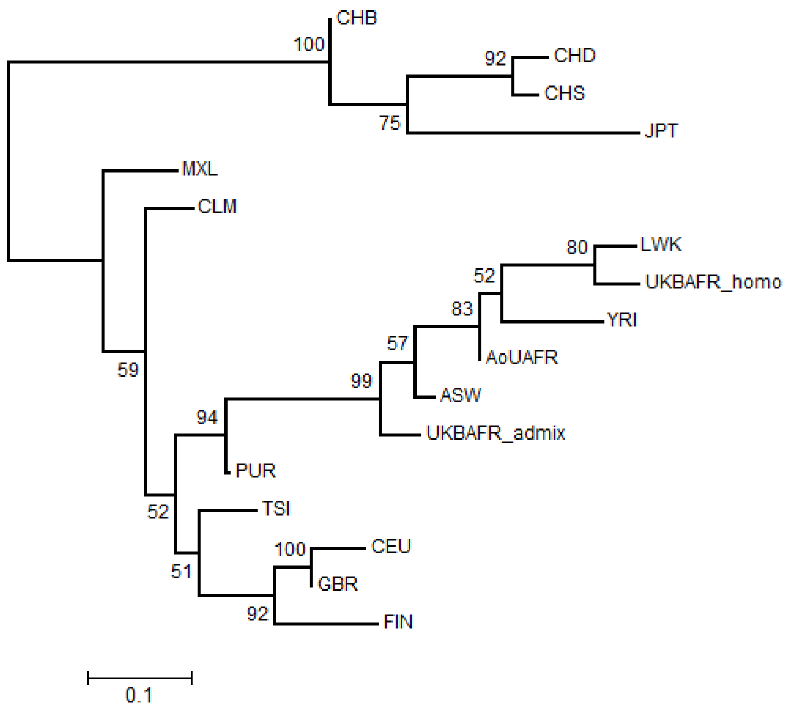

N-J phylogenetic tree among 1000G worldwide populations, UKB African homogenous subgroup (UKBAFR_homo, EUR proportion < 0.1, N=741), UKB African admixed subgroup (UKBAFR_admix, N=457), and AoU African populations (AoUAFR).

Figure S10

Figure S10. Pairwise linkage disequilibrium (LD) of HLA genotypes in UK Biobank African populations and its subgroups

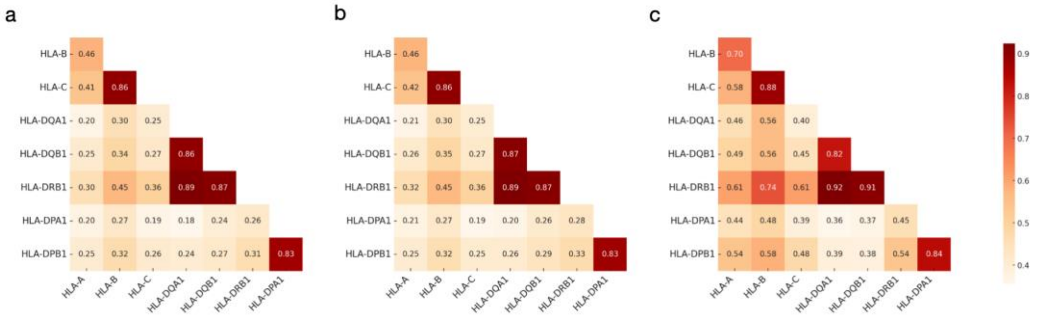

The pairwise linkage disequilibrium (LD) of HLA genotypes in UK Biobank African populations: all samples (a), Afr/Afr homogeneous group (b), and admixed group (c).

List of Supplemental Tables

Table S0 Resources (data/software) that have been used in this study

Table S1 The ethnic group code in the 1000 Genomes Project and H3Africa Consortium

Table S2 Number of unique second field HLA alleles in the UK Biobank African populations

Table S3 Comparisons of first field HLA typing genotypes in the UK Biobank African populations

Table S4 Comparisons of first field HLA tying genotypes in the 1000G African populations

Table S5 Comparisons of second field HLA tying genotypes in the 1000G African populations

Table S6 Genetic distance matrix among populations from UK Biobank and All of Us African and 1000G worldwide populations

Table S7 Frequencies of second field HLA genotypes in the UK Biobank African populations

Table S8 Results of exact test for deviations from HWE (Hardy-Weinberg equilibrium) test in the UK Biobank African populations

Table S9 Results of EWH (Ewens-Watterson homozygosity test of neutrality) in the UK in the UK Biobank African populations

Table S10 Pairwise linkage disequilibrium (LD) statistics of HLA genotype in the UK Biobank African populations

Table S11 Frequencies of four-digit HLA genotypes in the UK Biobank African homogeneous subgroup

Table S12 Results of exact test for deviations from HWE (Hardy-Weinberg equilibrium) test in the UK Biobank African homogeneous subgroup

Table S13 Results of EWH (Ewens-Watterson homozygosity test of neutrality) in the UK in the UK Biobank African homogeneous subgroup

Table S14 Pairwise linkage disequilibrium (LD) statistics of HLA genotype in the UK Biobank homogeneous subgroup

Table S15 Frequencies of haplotypes in the UK Biobank African populations

Table S16 Frequencies of haplotypes in the UK Biobank African homogeneous subgroup

Table S17 Frequencies of most common alleles in UKB, AFND database, and Anthony Nolan register, and reported associations in PharmGKB

Table S18 Frequencies of common haplotypesidentified in UKB across populations in the AFND database

Supplemental Material and Methods

Auxiliary Dataset 1: H3Africa dataset

The Human Heredity and Health in Africa (H3Africa) Consortium was set mainly to characterize genetic diversity across Africa for understanding the genetic background and health research of Africa. It provides unique opportunities for different projects of human disease across countries in Africa. In total 426 individuals from ongoing H3Africa studies underwent whole-genome sequencing (WGS), encompassing 50 ethnolinguistic groups from 13 African countries. In this study, we selected genetic data from two group to supplement the underrepresentation of African populations in the 1000 Genomes Project. This approach allows for a more comprehensive exploration of the genetic characteristics of African individuals in the UK Biobank.

Those two groups used in this study are Multiethnic from Botswana (BOT, N=46) and Bantu and Bantoid Speakers from Cameroon (CAM, N=50), representative of southern Africa and central Africa regions, respectively. WGS data are available through the European Genome-phenome Archive (EGA) under study accession number: EGAS00001002976. The data include genomic (BAMs and VCFs) and minimal phenotypic data from appropriately consented individuals. The details about sequencing platforms and data processing were listed in their publications (1). The approximate sequencing depth is 30×.

Auxiliary Dataset 2: All of US dataset

The All of Us dataset comprised a diverse group of US populations, especially under-represented demographic groups. The detailed information on recruitment and data description can be found in their publications (2). We randomly selected 983 African individuals and used their whole-genome sequencing data from controlled tier data available on the AoU Researcher Workbench, a comprehensive biomedical platform for data storage and analysis. Briefly, the WGS libraries were constructed with the Illumina Kapa HyperPrep kit and pooled and sequenced on the Illumina NovaSeq 6000 instrument. The initial quality control analysis was performed with the Illumina DRAGEN pipeline. The All of Us Research Program Genomics Investigators also performed joint variant calling across the entire All of Us WGS dataset and annotated using Illumina Nirvana. The above details were seen in their genomic publication (3).

HLA*LA: direct calling from whole genome sequencing in UKB and 10000G

The HLA*LA[(4)] ('linear alignments') is a novel graph-based method for the HLA type inference. It mainly implements the projections of linear alignments onto a variation graph and the likelihood model of HLA*PRG (5), which enables higher accuracy. The modified reference genome provided by the HLA*LA consists of GRCh38 plus the eight MHC haplotypes and IMGT genomic sequences (IPD-IMGT/HLA database, version 3.32.0) [(6)] .

We applied whole genome sequencing data to infer the 3-field HLA genotypes of UKB African individuals, using default options of the HLA*LA software on the

DNAnexus, the cloud-based UK Biobank Research Analysis Platform. Then, we extracted the “best guess” allele at G group resolution (exons 2/3 for class I genes, exon 2 for class II genes) inferring from individual WGS CRAM file. The “best guess” means that the genotype with the highest probability based on the likelihood-based inference procedure [(5)]. Additionally, it cost 20-35 mins per sample using the distance “mem2_ssd1_v2_x16” (16 Cores, 64 GB memory, 600 GB storage) on the DNAnexus platform. We applied the same data processing steps to 100 individuals from the 1000 Genomes Project dataset.

Michigan Imputation Server: imputation from assay genotypes in UKB and 10000G

The Michigan imputation server (MIS) [(7)] provides the HLA Imputation Pipeline using the imputation software Minimac4 within and four-digit multi-ethnic HLA v2 reference (2022, n=20,349) (8). This method has been well-recognized and widely used in the HLA imputation. For both UKB and 1000G populations, we extracted 10,456 and 9,638 SNPs on the MHC region, respectively. Then, we conducted initial quality control on assay genotyping data following the official document of MIS (https://imputationserver.readthedocs.io/en/latest /prepare-your-data/). The phasing and HLA genotype imputation were conducted, using the four-digit multi-ethnic HLA reference panel (v2) in the MIS. We extracted the classic two-digit and four-digit HLA allele genotype from the imputed genotype file, for the further comparison.

UKB-HLA: imputation using HLA*IMP:02 provided by UKB

The HLA genotypes (UK Biobank Data-field 22182) provided from the UK Biobank was carried out using HLA*IMP:02, and a multi-population reference panel was used. The imputation was based on SNP genotyping array data. A total of 211 HLA class I alleles, of which 53 are in the *HLA-A* locus, 126 are in the *HLA-B* locus, 32 are in the *HLA-C* locus, and a total of 135 HLA class II alleles, of which 59 are in the *HLA-DRB1* locus, 18 are in the *HLA-DQB1* locus, 14 are in the *HLA-DQA1* locus, 36 are in the *HLA-DPB1* locus, 8 are in the *HLA-DPA1* locus were included. The alleles with imputation posterior probability less than 0.8 were set to 0, and the allele '9901' indicates that no allele is present. For each HLA locus, the successful genotyping rate is over 97.7% (9). These data were widely used in HLA associations in the UK Biobank (10).

Kourami: direct calling from whole genome sequencing in All of Us

Based on the accessibility of computational resources and cost-effectiveness, we chose the Kourami as the HLA calling software for All of Us data. Kourami is a recently developed enrichment-free computational method that uses WGS data to directly assemble full sequences of the peptide-binding domain (exons 2 and 3 for class I human leukocyte antigen (HLA) genes and exon 2 for class II HLA genes) (11). The bam files of WGS data from 983 individuals were utilized to align to the preformatted IMGT-HLA database (Kourami panel). The Kourami constructed a combined multiple sequence alignment for each HLA locus, which we further modified by alignment projection. Using the weighted graphs with alignment paths,

candidate HLA alleles were identified by the best paths with the maximum weights (number of reads) and most supportive phasing information. We calculated the frequencies of four-digit HLA allele genotype for the construction of phylogenetic tree.

The ADMIXTURE analysis in UKB African population

To better explore the genetic ancestry components of the UKB African population, we used multiple combinations of representative population as reference group in the supervised ADMIXTURE analysis. First, we aim to determine which regional African population the genetic components of UKB African population most closely related to. We selected four African populations from different regions. Specifically, we included the LWK (Luhya in Webuye, Kenya) population representing Eastern Africa and the YRI (Yoruba in Ibadan, Nigeria) population representing Western Africa from the 1000 Genomes Project. Additionally, two populations were selected from the H3Africa dataset: CAM (Bantu and Bantoid Speakers from Cameroon) representing Central Africa and BOT (Multiethnic from Botswana) representing Southern Africa. The bar plot and density plot of genetic ancestry component in the ADMIXTURE analysis were displayed in the Figure S2.

Based on the optimal number (K = 3) of unsupervised ADMIXTURE analysis, we chose the two main components of four African populations in the further analysis, including LWK and YRI groups. Combing the CEU group representing European ancestry from the 1000G, we conducted the supervised ADMIXTURE analysis. To

enhance our analysis, we combined these three groups in pairs as references for the supervised ADMIXTURE analysis. The bar plot and density plot of genetic ancestry component in the ADMIXTURE analysis were displayed in the Figure 1. To enhance the representation of genetic diversity and ancestry proportions, we conducted pairwise calculations between above three groups. The bar plot and density plot of pairwise genetic ancestry component in the ADMIXTURE analysis were also displayed in the Figure S3-5.

Sensitivity analysis: global and local ancestry proportion correlation in UKB African population

To balance the need for genomic diversity and computational feasibility, we randomly selected five genomic regions from different chromosomes, each with a similar length (~ 5 Mb) to the HLA region. We then extracted the SNPs from those regions and performed local ancestry inference using the same RFMix2 pipeline applied in our main analysis for the HLA region. We also performed a similar correlation analysis by comparing the African ancestry proportions estimates from each genomic region to the genome-wide estimates at the individual level.

Sensitivity analysis: the ancestry proportion threshold in UKB African population

Based on the local ancestry inference of these segments, we calculated the ancestry proportions from the MHC region. Besides the threshold of 0.2, we tested the threshold of 0.1 and 0.3 for the European ancestry (CEU) proportion, categorizing

UKB African individuals into a homogeneous subgroup (African ancestry proportion > 0.9/0.7) and an admixed subgroup (European ancestry proportion > 0.1/0.3). To pinpoint the threshold for subgroups, we compared our second field genotype with the five-locus Sanger genotype of fourteen worldwide representative ethnic groups from 1000G, and AoU. The five-locus HLA allele frequencies from these populations were used to estimate genetic distance and construct a phylogenetic tree, used the same parameters and software as the Method part.

Reference

1. Choudhury A, Aron S, Botigue LR, Sengupta D, Botha G, Bensellak T, et al. High-depth African genomes inform human migration and health. Nature. 2020;586(7831):741-8.
2. All of Us Research Program I, Denny JC, Rutter JL, Goldstein DB, Philippakis A, Smoller JW, et al. The "All of Us" Research Program. N Engl J Med. 2019;381(7):668-76.
3. Bick AG, Metcalf GA, Mayo KR, Lichtenstein L, Rura S, Carroll RJ, et al. Genomic data in the All of Us Research Program. Nature. 2024;627(8003):340-6.
4. Dilthey AT, Mentzer AJ, Carapito R, Cutland C, Cereb N, Madhi SA, et al. HLA*LA-HLA typing from linearly projected graph alignments. Bioinformatics. 2019;35(21):4394-6.
5. Dilthey AT, Gourraud PA, Mentzer AJ, Cereb N, Iqbal Z, McVean G. High-Accuracy HLA Type Inference from Whole-Genome Sequencing Data Using Population Reference Graphs. PLoS Comput Biol. 2016;12(10):e1005151.
6. Robinson J, Barker DJ, Georgiou X, Cooper MA, Flicek P, Marsh SGE. IPD-IMGT/HLA Database. Nucleic Acids Res. 2020;48(D1):D948-D55.
7. Das S, Forer L, Schonherr S, Sidore C, Locke AE, Kwong A, et al. Next-generation genotype imputation service and methods. Nat Genet. 2016;48(10):1284-7.
8. Luo Y, Kanai M, Choi W, Li X, Sakaue S, Yamamoto K, et al. A high-resolution HLA reference panel capturing global population diversity enables multi-ancestry fine-mapping in HIV host response. Nat Genet. 2021;53(10):1504-16.
9. Bycroft C, Freeman C, Petkova D, Band G, Elliott LT, Sharp K, et al. The UK Biobank resource with deep phenotyping and genomic data. Nature. 2018;562(7726):203-9.
10. Wang QL, Wang TM, Deng CM, Zhang WL, He YQ, Xue WQ, et al. Association of HLA diversity with the risk of 25 cancers in the UK Biobank. EBioMedicine. 2023;92:104588.
11. Lee H, Kingsford C. Kourami: graph-guided assembly for novel human leukocyte antigen allele discovery. Genome Biol. 2018;19(1):16.
